# A Bat-Inspired Sparse Recovery Algorithm for Compressed Sensing

**DOI:** 10.1155/2018/1365747

**Published:** 2018-10-29

**Authors:** Wanning Bao, Haiqiang Liu, Dongbo Huang, Qianqian Hua, Gang Hua

**Affiliations:** China University of Mining and Technology, Xuzhou 221116, China

## Abstract

Compressed sensing (CS) is an important research area of signal sampling and compression, and the essence of signal recovery in CS is an optimization problem of solving the underdetermined system of equations. Greedy pursuit algorithms are widely used to solve this problem. They have low computational complexity; however, their recovery performance is limited. In this paper, an intelligence recovery algorithm is proposed by combining the Bat Algorithm (BA) and the pruning technique in subspace pursuit. Experimental results illustrate that the proposed algorithm has better recovery performance than greedy pursuit algorithms. Moreover, applied to the microseismic monitoring system, the BA can recover the signal well.

## 1. Introduction

In recent years, the collected data quantity by the Internet of things (IoT) has increased dramatically. To facilitate storage and transmission, signal sampling based on the traditional Shannon–Nyquist sampling theory works as follows: massive data are collected at the sampling stage and most are discarded at the compression stage. This process is extremely wasteful. CS, which was proposed by D. Donoho, E. Candès, and T. Tao, brings a revolutionary breakthrough in signal sampling [1–3]. If only *K* nonzero elements exist in a signal, the signal is called *K*-sparse signal with sparsity *K*. CS theory indicates that sparse signals can be recovered more accurately using less measurements than the Nyquist sampling principle.

The signal recovery problem in CS is a nonconvex combinatorial optimization problem. It can be solved by three types of algorithms. The first type is greedy pursuit algorithms, including Matching Pursuit (MP), Orthogonal Matching Pursuit (OMP) [4], Generalized Orthogonal Matching Pursuit (GOMP) [5], and Subspace Pursuit (SP) [6]. They are two-stage algorithms. In the first stage, they seek support iteratively. In the second stage, the signal can be recovered by using the least square method. This kind of algorithm is effective; however, they have weak recovery performance. The second kind of algorithm is a convex optimization algorithm, which recovers signals by converting nonconvex problems to convex problems. The most common convex optimization algorithms are Basic Pursuit (BP) and Gradient Projection for Sparse Reconstruction (GPSR) [7]. Although they have high accuracy, their computational complexity is high. The third kind of recovery algorithm is Bayesian algorithms [8], which can be divided into the following two types: Maximum A Posteriori (MAP) Estimation and Hierarchical Bayesian. The former underlines a signal distribution, and the latter introduces one or two variables, which control the sparse signal. Bayesian algorithms achieve a balance of high accuracy and short recovery time [9, 10]. In recent years, some scholars have applied Swarm Intelligence Algorithms to signal recovery in CS, such as Particle Swarm Optimization (PSO) [11] and the Grey Wolf Optimizer Algorithm [12]. These methods have good global search ability. However, they have a slow convergence velocity and are easy to be trapped into local optimum.

Recently, Yang et al. simulated bat echolocation and proposed a Bat Algorithm (BA) [13] based on stochastic optimization, which has simple structure, fast convergence, strong robustness, and parallel computing. The BA combines the advantages of PSO, Harmony Search (HS), Simulated Annealing (SA), and other algorithms. Many scholars applied it to optimization problems, such as numerical optimization, economic dispatch with random wind power [14], multirobot formation reconfiguration [15], microgrid operation management [16], and image processing [17]. In these areas, the BA shows better performance than do other intelligence optimizer algorithms.

In this paper, inspired by the BA and the greedy pursuit algorithm, an intelligence recovery algorithm for CS is proposed. OMP is used to initialize the swarm. The BA and the pruning technique are combined to update populations. The proposed algorithm outperforms the traditional greedy pursuit algorithms. Moreover, it runs faster than other swarm intelligence algorithms such as PSO.

The remainder of this paper is organized as follows: Section 2 briefly introduces the basic theory of CS and the BA. In Section 3, the proposed algorithm is introduced in detail. In Section 4, experiments are done to evaluate the algorithm performance. Finally, conclusions are drawn in Section 5.

The notations used in the rest of this paper include the following: |·| denotes the absolute value of a real number or the cardinality of a set. ‖·‖_*p*_(*p*=0,1,2,…) represents *l*_*p*_ − norm. For the vector *x* ∈ *R*^*n*^, $\hat{x}$ is the estimate of *x*. In a matrix *A* ∈ *R*^*m*×*n*^, *a*_*j*_ is the *j*-th column of *A*. *E*={1 ≤ *i* ≤ *n*|*x*_*i*_ ≠ 0}, which denotes the positions of nonzero elements in *x*, and it is called support set. *S*={1,2, ⋯, *n*} is the universal set of support. The matrix *A*_*E*_={*a*_*j*_}_*j*∈*E*⊆*S*_ is submatrix of *A*. The pseudoinverse of a matrix *A* is defined as *A*^+^=(*A*^*T*^*A*)^−1^*A*^*T*^, where *T* denotes the transpose operator.

## 2. Basic Theory

### 2.1. Compressed Sensing

Supposing an *n*-dimensional signal *y*=(*y*_1_, *y*_2_,…,*y*_*n*_)^*T*^ can be represented on a basis Ψ ∈ *R*^*n*×*n*^ as *y*=Ψ*x*, where *x*=(*x*_1_, *x*_2_,…,*x*_*n*_)^*T*^, if there are only *K* nonzero elements in *x*=(*x*_1_, *x*_2_,…,*x*_*n*_)^*T*^, *y* is called *K* − sparse signal with sparsity *K*, and the positions of nonzero elements in *x* are called the support set, which can be denoted as *E*={1 ≤ *i* ≤ *n*|*x*_*i*_ ≠ 0}. The signal *y* can be sampled by projection onto the measurement matrix Φ ∈ *R*^*m*×*n*^(*m* ≪ *n*) as

(1) $$\begin{array}{c} b=\Phi y=\Phi \Psi x=Ax, \end{array}$$

where *b*=(*b*_1_, *b*_2_,…,*b*_*m*_)^*T*^ is the measurement signal, *y* is the original signal, *x* is the sparse signal, Φ is the measurement matrix, and Ψ is the sparse basis. *A* ∈ *R*^*m*×*n*^ is the sensing matrix.

It can be seen that the data quantity is greatly reduced because *b*=Φy is an underdetermined system of equations. There are infinitely many solutions to recover *y* from *b*. However, if *y* is sparse, it can be recovered from *b* by solving the following *l*_0_ minimization problem:

(2) $$\begin{array}{c} \min_{x}\;{\left\|x\right\|}_{0},\\ \text{s}.\text{t}.\;Ax=b, \end{array}$$

where ‖·‖_0_ represents *l*_0_ − norm.

### 2.2. Orthogonal Matching Pursuit (OMP)

In the proposed algorithm, we use OMP to determine the initial solution.

The OMP algorithm can be stated in Algorithm 1 [4].

### 2.3. Bat Algorithm

In 2010, Yang et al. simulated the characteristics of bats' predatory behaviors and proposed BA [13, 18], which is a new Swarm Intelligence Optimization Algorithm.

The first step is randomly initializing the bat positions *x*_*i*_(0) and the velocities *v*_*i*_(0). Then, the BA updates the velocities and positions according to certain strategies. The velocities and positions of the *i*-th bat at *t*+1 iteration are updated as follows:

(3) $$\begin{array}{c} f_{i}=f_{\min}+\left(f_{\max}+f_{\min}\right)\beta , \end{array}$$

(4) $$\begin{array}{c} v_{i}\left(t+1\right)=v_{i}\left(t\right)+\left(x_{i}\left(t\right)+x_{*}\right)\cdot f_{i}, \end{array}$$

(5) $$\begin{array}{c} x_{i}\left(t+1\right)=x_{i}\left(t\right)+v_{i}\left(t+1\right), \end{array}$$

where *f*_*i*_ is the pulse frequency of the *i*-th bat, *f*_*i*_ ∈ [*f*_min_, *f*_max_], *β* ∈ [0,1] is a random number. *x*_*i*_(*t*) and *x*_*i*_(*t*+1) represent the positions of the *i*-th bat at the *t* and the *t*+1 iteration, respectively; *v*_*i*_(*t*) and *v*_*i*_(*t*+1) are the velocities of the *i*-th bat at the *t* and the *t*+1 iteration, respectively; and *x*_*∗*_ denotes the global best position of the swarm.

For each bat, the algorithm generates a random number *rand*1 ∈ [0,1]. If *rand*1 > *r*_*i*_(*t*), the position of this bat is updated according to formula (6), where *r*_*i*_(*t*) is the rate of pulse emission. The random walk is regarded as a procedure of the local search [13].

(6) $$\begin{array}{c} x_{i}\left(t+1\right)=x_{j}^{*}+\varepsilon L\left(t\right), \end{array}$$

where *x*_*j*_^*∗*^ is a random solution in the best solution set composed of the best solution of each bat, *ε* ∈ [−1,1] is a random number, and *L*(*t*) is the mean of the pulse loudness in the *t*-th iteration.

In addition, with the iterative number increasing, the pulse loudness *L*_*i*_ and the rate of pulse emission *r*_*i*_ need to be updated. During prey searching, the pulse loudness *L*_*i*_ will gradually decline, and the rate of pulse emission *r*_*i*_ will also gradually increase. The updating formulas of *L*_*i*_ and *r*_*i*_ are found in formulas (7) and (8), respectively:

(7) $$\begin{array}{c} L_{i}\left(t+1\right)=\alpha L_{i}\left(t\right), \end{array}$$

(8) $$\begin{array}{c} r_{i}\left(t+1\right)=r_{i}\left(0\right)\left[1-\exp\left(-\gamma t\right)\right], \end{array}$$

where *α* and *γ* are constants, 0 < *α* < 1, and 0 < *γ*.

The BA algorithm can be described in Algorithm 2 [13].

## 3. A Bat-Inspired Sparse Recovery Algorithm

### 3.1. Fitness Function

If the sparsity level *K* satisfying *K* < spark(*A*) is known as a priori, problem (2) can be approximated as follows [11, 12]:

(9) $$\begin{array}{c} {\mathrm{argmin}}_{x}\;{\left\|Ax-b\right\|}_{2},\\ \text{s}.\text{t}.\;\left\|x_{0}\right\|\leq K, \end{array}$$

where *b* is the measurement signal, and *A* ∈ *R*^*m*×*n*^ is the sensing matrix.

From greedy pursuit algorithms, we can see that the signal can be recovered by using a two-step strategy. First, estimate the support set and then estimate the signal using the least square method.

(10) $$\begin{array}{c} x_{\hat{E}}=A_{\hat{E}}^{+}b,\\ x_{S-\hat{E}}=0, \end{array}$$

where $\hat{E}$ denotes the estimated support set and *S*={1,2, ⋯, *n*}. The vector *x*_*E*_ consists of the entries of *x* ∈ *R*^*n*^ indexed by *i* ∈ *E* ⊆ *S*. The matrix *A*_*E*_={*a*_*j*_}_*j*∈*E*⊆*S*_ is the submatrix of *A*, and *a*_*j*_ is the *j*-th column of *A*. *A*^+^=(*A*^*T*^*A*)^−1^*A*^*T*^ is the pseudoinverse of a matrix *A*, where *T* denotes the transpose operator.

Once the support set is estimated accurately, the following equation must be satisfied.

(11) $$\begin{array}{c} {\left\|A_{\hat{E}}A_{\hat{E}}^{+}b-b\right\|}_{2}=0, \end{array}$$

where ‖·‖_2_ represents *l*_2_ − norm.

Because ${\left\|A_{\hat{E}}A_{\hat{E}}^{+}b-b\right\|}_{2}\geq 0$, we define the fitness function as

(12) $$\begin{array}{c} f\left(\hat{E}\right)={\left\|A_{\hat{E}}A_{\hat{E}}^{+}b-b\right\|}_{2}. \end{array}$$

### 3.2. Initialization

Suppose there are *I* bats, the position of the first bat is initialized as the estimated support set of the OMP. For the *i*-th (2 ≤ *i* ≤ *I*) bat, its position is initialized as follows.

Randomly choose *q*=0.8 × *m* elements from the set *S*={1,2,…, *n*} and form the set *V*_*i*_(0); here, *V*_*i*_(*t*) is regarded as the velocity of the *i*-th bat at the *t* iteration. Then, the position of the *i*-th (2 ≤ *i* ≤ *I*) bat is a set formed by *K* indices corresponding to the maximum absolute values in *A*_*V*_*i*_(0)_^+^ · *b*. Other related parameters are initialized as follows: the pulsing frequency *f*_*i*_ of each bat is initialized according to formula (3), where *f*_min_=0.8and*f*_max_=1. The initial emission rate *r*_*i*_(0) ∈ [0,1]. The pulse loudness *L*_*i*_(0) ∈ [0,1], *β* is a random number between 0 and 1, and *ε* is a random number between −1 and 1, 0 < *α* < 1, and 0 < *γ*.

### 3.3. Update Strategy

The main innovation of our algorithm is the update strategy. The iterative process combines the thought of BA and greedy pursuit algorithm. The update strategy can be divided into three parts.

The first part is the update of velocity and position: a set *Q*_*i*_(*t*) is formed with *β* · *f*_*i*_ · *K* elements selected randomly from the best solution *E*_*∗*_; we define *U*_*i*_(*t*)=*E*_*i*_(*t*) ∪ *Q*_*i*_(*t*). If |*U*_*i*_(*t*)| ≤ *q*, a set *G*_*i*_(*t*) consists of *q* − |*U*_*i*_(*t*)| elements selected randomly from the set *S* − *U*_*i*_(*t*), update *V*_*i*_(*t*+1)=*U*_*i*_(*t*) ∪ *G*_*i*_(*t*). If |*U*_*i*_(*t*)| > *q*, the set *G*_*i*_(*t*) consists of *q* − *K* elements selected randomly from the set *Q*_*i*_(*t*) − *E*_*i*_(*t*), update *V*_*i*_(*t*+1)=*E*_*i*_(*t*) ∪ *G*_*i*_(*t*). The position of the *i*-th bat *E*_*i*_(*t*+1) is a set composed of the indices corresponding to the *K* maximum absolute values in *A*_*V*_*i*_(*t*+1)_^+^ · *b*.

The second part is the local search: generate a random number *rand*1 ∈ [0,1]; if *rand*1 > *r*_*i*_(*t*), select a random solution *E*_*j*_^*∗*^ from the best solutions set and update the position of this bat by randomly replacing *ε* · *L*_*i*_(*t*) · *K* elements of this solution with other elements in *S* − *E*_*j*_^*∗*^. After updating all positions, calculate the new fitness *f*(*E*_*i*_(*t*+1)) of each bat. Specifically, in order to accelerate the convergence speed of this algorithm, all the new solutions will be accepted in the algorithm.

The third part is the adjustment of the emission rate *r*_*i*_(*t*) and the pulse loudness *L*_*i*_(*t*) as formulas (7) and (8). If *f*(*E*_*i*_(*t*+1)) < *f*(*E*_*i*_^*∗*^), update the best solutions set *E*_*i*_^*∗*^=*E*_*i*_(*t*+1). If *f*(*E*_*i*_(*t*+1)) < *f*(*E*_*∗*_), update the global best solution *E*_*∗*_=*E*_*i*_(*t*+1).

### 3.4. Stopping Criterion

If $f\left(\hat{E}\right)<\sigma$, the iteration is terminated; here, *σ* is the termination threshold. Moreover, in order to avoid too many iterations, set a maximum allowed iteration number *N*_*max*. If the iteration number is larger than *N*_*max*, the iteration is also terminated.

The algorithmic process in Algorithm 3.

### 3.5. Efficiency Analysis

The computational complexity of the algorithm is mainly dependent on the initialization and iteration.

The algorithm initializes solutions using OMP, which has the complexity *O*(*Kmn*) [19].

In each iteration, the complexity is mainly dependent on three operations. The first is the multiplication of matrix, which has the computational complexity *O*(*mn*). The second is sorting of an n-dimensional vector, which has the computational complexity *O*(*nlogn*). The third is the least square, which has the computational complexity *O*(*m*^3^). In summary, the computational complexity in the iteration phase is *O*(*Im*^3^+*Imn*+*Inlogn*), where *I* is the population size.

In general, the computational complexity of the proposed algorithm is similar to OMP when the signal sparsity is small. The complexity depends on the number of iterations when the sparsity is larger or when the measurement number is small. Moreover, this algorithm uses the idea of the BA, which combines the advantages of PSO, HS, SA, and other intelligence algorithms to optimize the convergence speed and search accuracy. It usually runs faster than other intelligence algorithms. OMP can generate an initial solution closed to the target, which accelerates the convergence of the BA. In addition, as swarm intelligence algorithm, it can run in parallel to reduce the running time [12, 20].

## 4. Experiments

In this section, we compare this algorithm with OMP, GOMP, SP, Fast Laplace, and PSO, where Fast Laplace is a Bayesian-based algorithm, and PSO is a swarm intelligence algorithm for CS. Then, we apply this algorithm to recover the real microseismic signals.

Candes and Tao proved that the Gaussian random matrix [2, 21, 22] with independent identically distribution can be a universal measurement matrix. Therefore, the measurement matrices in these experiments are the Gaussian random matrix with the size of *m* × *n*. The number of iterations of the OMP, GOMP, and SP algorithms is *K*. The maximum number of iterations of PSO and the proposed algorithm are *N*_max_=100. The algorithm will stop when *f*(*E*^*∗*^) < *σ* or the number of iterations reaches *N*_*max*.

The software and hardware environment of the experiment is as follows:

  Processor: Intel (R) Pentium (R) CPU G3220 @ 3.00 GHz 3.0
  Memory: 4.00 G
  Operating system: 64-bit Windows7
  Simulation software: MATLAB R2014a

### 4.1. Comparison with Other Algorithms

In the first experiment, we compare the recovery performance of different algorithms against the change of sparsity. We use a 128 × 256 random Gaussian matrix to sample a 256-length sparse signal. The sparsity level is set as *K*=35, 40, 45, 50, 55, 60, 64, 70. The parameters are set as *n*=256, *m*=128, *σ*=1*e* − 8, *f*_min_=0.8,  and  *f*_max_=1; the rate of pulse emission *r*_*i*_(0) and the pulse loudness *L*_*i*_(0) are set between 0 and 1; *β*=0.2, *ε*=0.2, *α*=0.9, and *γ*=0.9. Here, we compare the algorithm performance with the following two metrics: recovery error and exact recovery rate.

**Figure 1:**
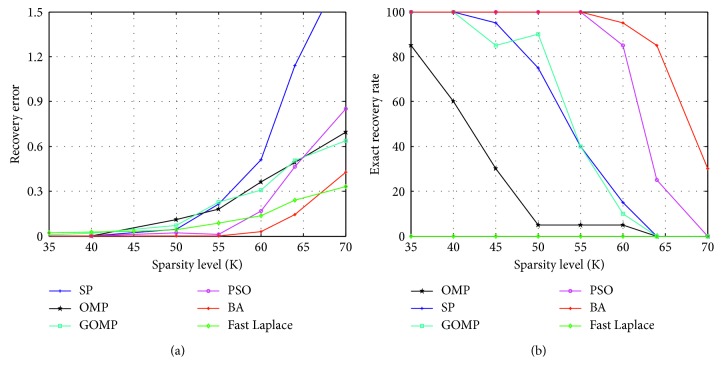
Comparison of the algorithm recovery performance against the change of sparsity with *n*=256, *m*=128, and the sparsity *K*=35, 40, 45, 50, 55, 60, 64, 70. (a) Recovery error. (b) Exact recovery rate.

Recovery error is a metric to evaluate the error between the original sparse signal and the recovered sparse signal. The recovery error is defined as follows (13):

(13) $$\begin{array}{c} \text{Recovery error}=\frac{1}{\text{CNT}}\overset{\text{CNT}}{\underset{j=1}{\sum }}\left(\frac{{\left\|x^{\left(j\right)}-{\hat{x}}^{\left(j\right)}\right\|}_{2}^{2}}{{\left\|x^{\left(j\right)}\right\|}_{2}^{2}}\right), \end{array}$$

where *x* is the original sparse signal, $\hat{x}$ is the recovered sparse signal, and CNT=100 is the cycle number to obtain the average performance. The simulation result of recovery error is shown in Figure 1(a). The exact recovery rate is obtained by calculating the number of times that the estimated values are exact. If $x-{\hat{x}}_{2}<10^{-6}$, this experiment is considered successful. In 100 experiments, if there are *s* successful experiments, the exact recovery rate is (*s*/100). The exact recovery rates of different algorithms are shown in Figure 1(b).

It can be seen that when the sparsity is large, the recovery errors of three commonly used greedy pursuit algorithms such as OMP, GOMP, and SP increase rapidly, and the exact recovery rate decreases quickly. The recovery error of the Fast Laplace algorithm is lower than that of traditional greedy pursuit algorithms. However, experiments demonstrate that the exact recovery rates of the Bayesian algorithm are 0 because there exists no experiment satisfying $x-{\hat{x}}_{2}<10^{-6}$. The swarm intelligence algorithms also perform well. The recovery error of the proposed algorithm is lower than 0.4, and the exact recovery rate is higher than other five algorithms when the sparsity is very large.

In the second experiment, we compare the recovery performances against the change of measurement number. We use a *m* × 256 Gaussian matrix to sample a 256-length signal with sparsity 30. The measurement number is set as *m*=53, 58, 63, 68, 78, 88, 98, 108, 118, and other parameters are the same to the first experiment.

**Figure 2:**
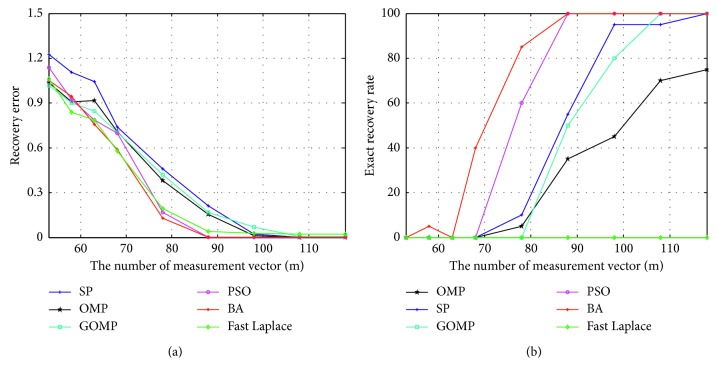
Comparison of the signal recovery performance against the change of measurement number with *n*=256, *m*=53, 58, 63, 68, 78, 88, 98, 108, 118 and sparsity *K*=30. (a) Recovery error. (b) Exact recovery rate.

Figures 2(a) and 2(b) illustrate the recovery error and the exact recovery rate, respectively, of the six algorithms under different measurement numbers. As shown in the figure, the recovery of the proposed algorithm performs better than those commonly used greedy pursuit algorithms such as OMP, GOMP, and SP, when the number of measurement vectors is small. It also has more advantages compared with the PSO algorithm and the Fast Laplace algorithm.

**Figure 3:**
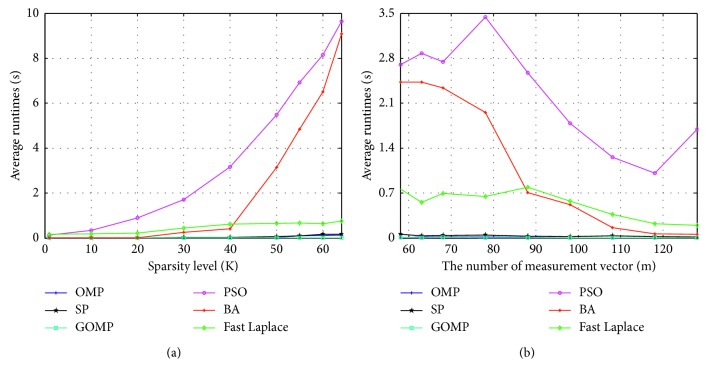
Comparison of average running times. (a) Running time of the six algorithms against the change of sparsity with *n*=256, *m*=128, and *K*=1, 10, 20, 30, 40, 50, 55, 60, 64. (b) Running time of the six algorithms against the change of measurement number with *n*=256, *K*=30, and *m*=53, 58, 63, 68, 78, 88, 98, 108, 118.

In the third experiment, we compare the recovery efficiency of different algorithms.  Figure 3(a) is the running time of the six algorithms with different sparsity. The sparsity level is set as *K*=1, 10, 20, 30, 40, 50, 55, 60, 64.  Figure 3(b) is the running time of the six algorithms with different measurement number. The measurement number is taken as *m*=58, 63, 68, 78, 88, 98, 108, 118, 128. Other parameters are the same as those of the first experiment, and the average time of each algorithm in recovering the signal is counted. As shown in Figure 3, when the sparsity is small or the measurement number is large, the recovery efficiency of this algorithm is similar to the commonly used greedy pursuit algorithms such as OMP. Although the running time of the algorithm increases when the sparsity is large or the measurement number is small, the algorithm is more efficient than other swarm intelligence algorithms such as PSO. However, as a swarm intelligence algorithm, it can run in parallel to reduce the running time [12, 20].

### 4.2. Application in Microseismic Signals

Microseismic monitoring is an effective technology for accident prediction in coal mines. Under the background of coal mine IoT, the collected data quantity by numerous microseismic nodes in the monitoring area are massive [23]. The effective compression of microseismic data can reduce the storage space and energy consumption. It plays an important role in improving the data transmission rate. Participants of the project collected three microseismic signals from a coal mine in Anhui Province, China. In this section, we use these signals as the original signal and apply the proposed algorithm to recover signal.

**Figure 4:**
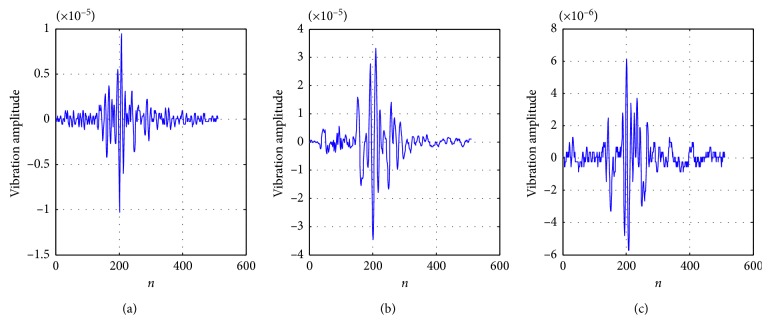
Original waveforms of the three microseismic signals: (a) signal 1, (b) signal 2, and (c) signal 3.

We perform experiments on three different microseismic signals. The length of the signal is *n*=512, as shown in Figure 4. It can be seen that the microseismic signals are one-dimensional time-frequency signals. Microseismic signals are not sparse in the time domain; however, their representations under a certain basis are sparse or compressible.

**Figure 5:**
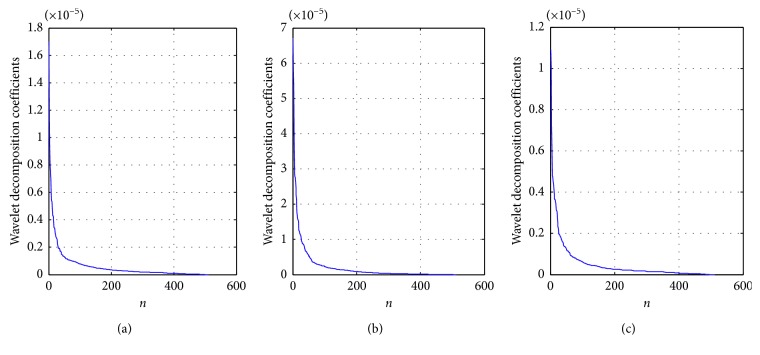
Wavelet decomposition coefficients of three microseismic signals: (a) signal 1, (b) signal 2, and (c) signal 3.

In this experiment, the 2-layer db2 wavelet transform [24] is used as sparse basis. Wavelet transform uses a cluster of functions to approximate a signal which can be seen as a decomposition of the signal. It divides the signal into a low-frequency signal and multiple high-frequency signals. The low-frequency signal is the stationary part of the signal. The high-frequency signal indicates the details of the signal.  Figure 5 shows the wavelet decomposition coefficients of three groups of microseismic signals.

**Figure 6:**
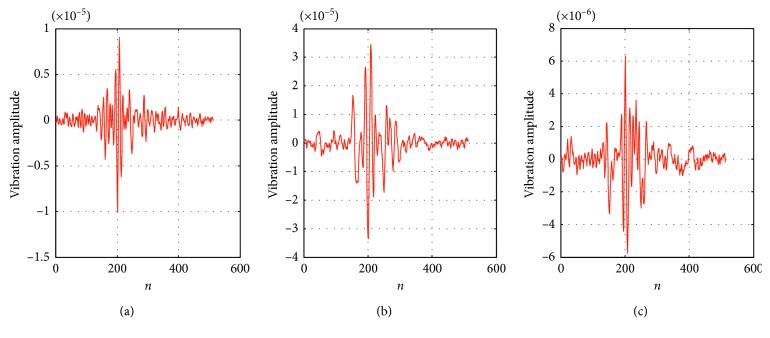
Recovered waveforms of the three microseismic signals: (a) signal 1, (b) signal 2, and (c) signal 3.

The measurement matrix used in this experiment is a 256 × 512 random Gaussian matrix. The sparsity level is *K*=128 and *σ*=1*e* − 5.  Figure 6 shows the three groups of recovered signals. The recovery errors in the three groups of experiments are 0.1862, 0.1250, and 0.1512.

## 5. Conclusions

In this paper, an Intelligence Sparse Recovery Algorithm is proposed for CS inspired by the BA and the pruning technique of SP. The algorithm inherits the global search ability and the local search ability of the BA, and we use OMP to obtain a better initial solution to accelerate the convergence. Compared with the traditional pursuit algorithm, the algorithm in this paper has better recovery performance under large sparsity and small measurement. At the same time, it runs faster than the intelligent optimization algorithm such as PSO. The proposed algorithm can be applied to the microseismic monitoring system, which can recover the signal well. In future research, performance and efficiency of the algorithm will be more rigorously analyzed.

## Figures and Tables

**Algorithm 1:**
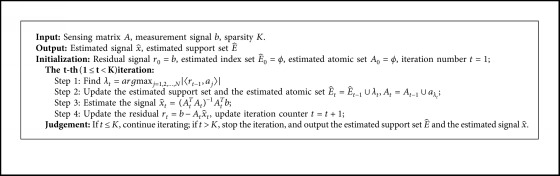
Orthogonal Matching Pursuit (OMP).

**Algorithm 2:**
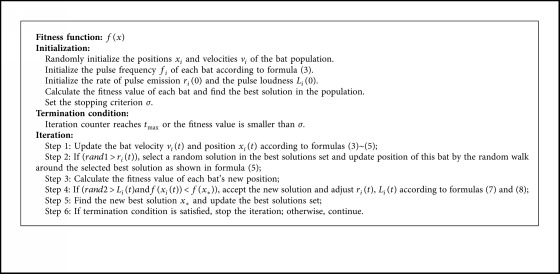
Bat Algorithm (BA).

**Algorithm 3:**
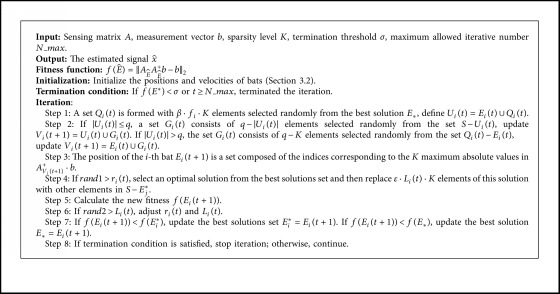
A Bat-Inspired Sparse Recovery Algorithm for CS.

## Data Availability

The data of microseismic signals used to support the finding of this study have not been made available for the time being because the data are obtained through cooperation between us and the Coal Mine Group, and it must be approved by both parties before it can be disclosed.
